# Characterization of *Paenibacillus* sp. GKG Endo-β-1, 3-Glucanase, a Member of Family 81 Glycoside Hydrolases

**DOI:** 10.3390/microorganisms10101930

**Published:** 2022-09-28

**Authors:** Gediminas Plakys, Renata Gasparavičiūtė, Justas Vaitekūnas, Rasa Rutkienė, Rolandas Meškys

**Affiliations:** 1 Department of Molecular Microbiology and Biotechnology, Institute of Biochemistry, Life Sciences Center, Vilnius University, Sauletekio 7, LT-10257 Vilnius, Lithuania; 2 R&D Department, Roquette Amilina, AB, J. Janonio 12, LT-35101 Panevezys, Lithuania

**Keywords:** endo-1,3-β-glucanase, *Paenibacillus*, GH81, CBM56, yeast cell walls

## Abstract

*Paenibacillus* sp. GKG was isolated based on its ability to produce hydrolysis zones on agar plates containing yeast cell wall substrate as the single carbon source. The extracellular enzymes secreted into the culture medium were identified by LC-MS/MS proteomics. Endo-β-1,3-glucanase PsLam81A containing GH81 catalytic and the CBM56 carbohydrate-binding modules was selected for heterologous expression in *Escherichia coli*. The identity of the recombinant PsLam81A was confirmed by LC-MS/MS proteomics. The PsLam81A showed the highest activity at 60 °C, and the optimal pH range was between 6.5 and 8.0. The analysis of the full-length PsLam81A and truncated PsLam81AΔCBM56 enzymes showed that the CBM56 module improved the hydrolytic activity towards linear β-1,3-glucans—curdlan and pachyman but had no effect on hydrolysis of β-1,3/β1,6-branched glucans—laminarin and yeast β-glucan. The characterization of PsLam81A enzyme broadens current knowledge on the biochemical properties and substrate specificity of family 81 glycoside hydrolases and allows prediction of the necessity of CBM56 module in the process of designing new truncated or chimeric glycosidases.

## 1. Introduction

A vast diversity of glycoside hydrolases (GHs) has been discovered so far. A group of GHs that catalyze the hydrolysis of β-1,3-D-glycosidic and in some cases β-1,4-D-glycosidic bonds is subdivided into three classes: exo-β-1,3-glucanases (EC 3.2.1.58), which cleave D-glucose from the nonreducing end of glucan molecule; endo-β-1,3-glucanases (EC 3.2.1.39), which require at least two β-1,3-bound glucose residues adjacent to the digested bond; and endo-β-1,3(4)-glucanases hydrolyzing both β-1,3 and β-1,4 bonds, as long as the glucose residue whose reducing group is involved in the linkage to be hydrolyzed is itself substituted at C-3 [1].

GHs are enzymes that participate in the breakdown of complex carbohydrates and are classified into families at the CAZy database according to amino acid sequences [2]. Endo-β-1,3-glucanases (EC 3.2.1.39) are found in 10 families: GH5, GH16, GH17, GH55, GH64, GH81, GH128, GH152, GH157, GH158. The majority of bacterial endo-β-1,3-glucanases belong to family GH16 and the majority of fungal endo-β-1,3-glucanases belong to family GH81 [1]. Endo-β-1,3-glucanase (EC. 3.2.1.39) activity is the only activity reported from members of family 81, so far. According to the CAZy database, the majority of characterized enzymes from the GH81 family is of fungal origin—there are only three bacterial enzymes characterized in this family [3,4,5]. GHs often are multimodular—besides catalytic module they may contain ancillary modules, for example, carbohydrate-binding modules (CBMs)—these modules improve the adhesion of enzymes to carbohydrates but have no catalytic activity themselves [6]. CBM56 module is reported to be present in the *Paenibacillus* genus (e.g., family 64 GH PbBgl64A from *Paenibacillus barengoltzii*) and in family 81 GH (e.g., BH0236 from *Bacillus halodurans* (*Halalkalibacterium halodurans*)) [5,7].

Laminarin is a water-soluble β-1,3-glucan with β-1,6 side chain branches majority of which are single glucosyl residues. Laminarin from *Laminaria digitata* has a heterogenous structure as it is comprised of the β-1,3-glucan backbone with a degree of polymerization (DP) ranging from 20 to 30 and with ratio of three-to-one between M-chain and G-chain molecules. M-chain molecules are terminated with nonreducing 1-linked D-mannitol, while G-chain molecules are terminated with reducing 3-linked glucose. One β-1,3 backbone can have from zero to four β-1,6 branches with an average of 1,3 branches per molecule [8]. Laminarin is water-soluble because of these short occasional branches. Unbranched laminari-oligosaccharides are freely water-soluble only if they have DP lower than 20. Unbranched laminari-oligosaccharides with DP higher than 36 (e.g., curdlan) are water-insoluble. The increase in β-1,3-backbone length (DP) or the increase in branches length decreases the solubility of β-1,3-glucan [9]. Therefore, yeast β-1,3-glucan that has an average DP of about 1500 and β-1,3 branches linked via β-1,6- bond is water-insoluble [9,10]. The yeast cell wall is composed of four main components: β-1,3-glucan (50–55%), β-1,6-glucan (5–10%), mannoprotein (35–40%) and chitin (1–2%) [11]. These components make-up a complicated water-insoluble yeast cell wall structure with various intra-chain and inter-chain bonds. Polysaccharides can be cross-linked via glycosidic bonds such as β-1,3-, β-1,4-, β-1,6-, α-1,2, α-1,4, α-1,6 or linked to proteins via amino or hydroxy groups of amino acid residues [12]. This trait makes yeast cell walls a broad spectrum substrate suitable for the screening of microorganisms producing various types of glycoside hydrolases based on the hydrolysis zone on solid agar plates. The appearance of hydrolysis zones can be expected in the presence of GHs such as β-1,3-glucanases, β-1,6-glucanases, α-1,2, α-1,4, α-1,6-mannanases and chitinases. The yeast cell walls treated with such GHs might have decreased DP of backbone, shorter length of branches and/or inter-chain bonds hydrolyzed, which makes substrate soluble. The polysaccharide substrates can be used as the only carbon-source in agar plates. For example, Avicel or CMC-Na were used as the carbon source during the screening for cellulolytic bacteria, hydrolysis zones were inspected after visualization using Congo red [13].

β-1,3-Glucanases have inspired an array of biotechnological applications including the conversion of lignocellulosic biomass into sugars, as clarification agents in winemaking, as feed additives or as biocontrol agents against pathogenic fungus in various crops [1]. The advances of next-generation sequencing technologies enabled the continuous growth in a number of gene sequences deposited into the CAZy database; meanwhile, the number of biochemically or structurally characterized CAZymes is growing at a much slower rate. The biochemical characterization of enzymes identified during sequencing genomes of microorganisms is important because of the advanced properties that newly discovered enzymes can offer to industrial applications. Cold-adaptivity and high thermal stability are examples of industrially relevant traits these enzymes can have [14,15]. Enzymatic hydrolysis has been extensively applied for the biomass hydrolysis and valorization of various industrial by-products. Yeast cell walls are generally considered a low-value by-product acquired during the production of yeast extracts [16]. Since complete disruption of yeast cell walls by enzymatic hydrolysis requires various enzymatic activities, the yeast cell walls themselves are an excellent substrate for screening such enzymes-producing microorganisms.

In this study, we have reported the isolation of *Paenibacillus* sp. GKG bacterium that is able to hydrolyze polysaccharides from yeast cell walls. We successfully identified and cloned the β-glucanase gene of the GH81 family responsible for this activity. The production and characterization of recombinant endo-β-1,3-glucanase revealed the substrate specificity of this enzyme and the mode of action. This work demonstrates the workflow suitable for screening new GHs using yeast cell wall polysaccharides as a substrate and broadens the current knowledge about members of the GH81 family.

## 2. Materials and Methods

### 2.1. Substrates and Chemical Reagents

Laminari-oligosaccharides, curdlan, pachyman, barley β-glucan, yeast β-glucan, sugar beet β-glucan were purchased from Megazyme (Wicklow, Ireland). Laminarin, carboxymethyl cellulose and microcrystalline cellulose were purchased from Sigma-Aldrich (St. Louis, MO, USA). Pustulan was purchased from Biosynth-Carbosynth (Bratislava, Slovakia). Additional information about the substrates used in this study can be found in Table 1.

Ammonium bicarbonate (ABC), clotrimazole, 3,5-dinitrosalicylic acid (DNS), DL-dithiothreitol (DTT), iodoacetamide, imidazole, p-anisaldehyde, triton X-100 and urea were purchased from Sigma-Aldrich (St. Louis, MO, USA). Acetonitrile (LC-MS grade) and formic acid (LC-MS grade) were purchased from Chem-Lab NV (Zedelgem, Belgium). Glycine and ortho-phosphoric acid (85%) were purchased from Merck (Darmstadt, Germany). Citric acid monohydrate, HCl (35–38%), NaCl and NaOH were purchased from ChemPur (Piekary Slaskie, Poland). Acetic acid (99.8%), potassium sodium tartrate tetrahydrate and H_2_SO_4_ (96%) were purchased from Lach-Ner (Neratovice, Czech Republic). Ampicilin, ethylenediaminetetraacetic acid (EDTA), KCl, K_2_HPO_4_, Na_2_HPO_4_, trizma baze was purchased from Carl-Roth (Karlsruhe, Germany). Agarose tablets, isopropyl-β-D-thiogalactopyranoside (IPTG) and proteinase K were purchased from Thermo Fisher Scientific (Vilnius, Lithuania). KH_2_PO_4_ and lysozyme were purchased from Fluka (Buchs, Switzerland). Agar was purchased from Formedium (Hunstanton, UK). Ethanol (99.8%) was purchased from Honeywell (Seelze, Germany). Ethidium bromide was purchased from Calbiochem (San Diego, CA, USA). Butan-1-ol, NH_4_H_2_PO_4_ and MgSO_4_ × 7H_2_O were purchased from Reakhim (Moscow, Russia).

### 2.2. Substrate Preparation for Screening of Microorganisms

Compressed baker’s yeast (Lallemand, Lublin, Poland) suspension was prepared in reverse osmosis treated water at a concentration of 7.6 g (dry weight)/100 g of suspension. The suspension pH was 7.6 and it contained 0.7% NaHSO_3_ and 0.7% Na_2_CO_3_. Yeast cells were disrupted using high shear fluid processor microfluidizer M7250-10 (Microfluidics, Newton, MA, USA). The operating pressure of 690 bar was used and 8 passages were performed. The temperature of the effluent did not exceed 18 °C. The resulting suspension was centrifuged at 12,200× *g* for 10 min. The supernatant was discarded, the yeast cell debris (further in the text referred as cell walls) layer was removed and transferred to a new centrifuge bottle, remaining undisrupted yeast cell layer was discarded. The resulting cell wall fraction was washed with water: 2 parts of water (by weight) was added to a yeast cell wall fraction. The mixture was homogenized using a kitchen blender SHB 4460WH-EUE3 (Sencor, Ricany, Czech Republic). The resulting suspension was centrifuged at 12,200× *g* for 10 min, the supernatant was discarded, yeast cell wall layer was removed and transferred to a new centrifuge bottle. The procedure was repeated two more times. After washing with water, the yeast cell wall fraction was frozen at −80 °C and freeze-dried for 48 h at −48 °C using a freeze-dryer Modulyo D-115 (Thermo Savant, Waltham, MA, USA). Freeze-dried yeast cell wall fraction was ground using laboratory mill M20 (IKA, Staufen, Germany).

### 2.3. Isolation of Bacteria

Soil sample was collected from the pond located in the city of Vilnius, Lithuania (54.709437, 25.271094). The sample was enriched for yeast cell wall degrading microorganisms by adding yeast cell walls to a final concentration of 10 mg/mL and incubated at 22 °C for 120 h. Enriched samples were diluted 25-fold using 0.9% NaCl, then 50 µL aliquots were plated on a solid mineral medium containing a water-insoluble yeast cell wall substrate as a carbon source. The solid mineral medium contained 5 g/L NaCl, 1 g/L NH_4_H_2_PO_4_, 1 g/L K_2_HPO_4_, 2 g/L MgSO_4_ × 7H_2_O, 15 g/L agar, 3 g/L lyophilized yeast cell walls and 6 mg/L clotrimazole, pH 7.2. The plates were incubated at 30 °C for 72 h. Then the plates were visually inspected and colonies that were able to produce hydrolysis zones were isolated by repeated streaking.

### 2.4. Identification of Isolates

Chromosomal DNA from isolated colonies was extracted as described previously [17]. The 16S rRNA gene was amplified by PCR using chromosomal DNA as a template, Phusion Plus PCR Master Mix (Thermo Fisher Scientific, Vilnius, Lithuania) and primers W001 and W002 (Table S1) [18]. The PCR products were purified using GeneJET Gel Extraction Kit (Thermo Fisher Scientific, Vilnius, Lithuania). Purified PCR products were cloned into pJET1.2/blunt cloning vector using CloneJET PCR Cloning Kit (Thermo Fisher Scientific, Vilnius, Lithuania). *E. coli* strain DH5α (Fermentas, Vilnius, Lithuania) cells were transformed with ligation mixtures and spread on LB agar plates supplemented with 50 µg/mL ampicillin. pDNA was extracted and purified using the ZR Plasmid Miniprep kit (Zymo Research, Irvine, CA, USA) and sequenced using pJET1.2 sequencing primers (Table S1) by the Sanger method (Macrogen Europe, Amsterdam, The Netherlands).

### 2.5. Production of Hydrolytic Enzymes

The liquid mineral medium used for the production of extracellular enzymes contained 5 g/L NaCl, 1 g/L NH_4_H_2_PO_4_, 1 g/L K_2_HPO_4_, 2 g/L MgSO_4_ × 7H_2_O and 3 g/L lyophilized yeast cell walls, pH 7.2. A single colony from a solid mineral medium was inoculated in 20 mL of liquid mineral medium for 24 h at 30 °C 180 rpm. 10 mL of inoculum was transferred to 200 mL of liquid mineral medium and incubated for 48 h at 30 °C 180 rpm. The culture medium was centrifuged at 7200× *g* for 20 min at 4 °C. The supernatant was collected and pellet was discarded. The supernatant was concentrated 20-fold using Amicon Stirred Ultrafiltration Cell 8200 (Millipore, Burlington, MA, USA) equipped with Omega 76 mm 30K membrane discs (Pall Corporation, New York, NY, USA). Concentrated supernatant was dialyzed in a 14K dialysis membrane tubing (Carl-Roth, Karlsruhe, Germany) at 4 °C against 10 mM Tris-HCl, pH 7.0, for 18 h. Afterwards, the dialyzed samples were subjected to a tryptic digest.

### 2.6. Filter Aided Protein Sample Preparation (FASP) for Mass Spectrometry Analysis

FASP of protein samples was performed using Nanosep^®^, MWCO 10 kDa ultrafiltration centrifugal units (Pall Corporation, New York, NY, USA) at 13,000× *g*. Sample preparation and tryptic digestion were performed according to a modified FASP method described earlier [19]. The protein sample was mixed with denaturing buffer (8 M urea 100 mM ABC pH 8.0) in a ratio 1:7 (sample:denaturing buffer, v/v). Denaturing buffer was exchanged for 100 µL reducing buffer (10 mM DTT in denaturing buffer). The sample in reducing buffer was incubated in a Thermomixer C (Eppendorf, Hamburg, Germany) at 37 °C 600 rpm for 60 min. After incubation, 200 µL of denaturing buffer were added to centrifugal units, after centrifugation, flowthrough was discarded. 100 µL of alkylation buffer (50 mM iodoacetamide in denaturing buffer) were added and centrifugal units were incubated at 37 °C 600 rpm for 60 min. DTT was added to yield a final concentration of 50 mM to deactivate residual iodoacetamide. Alkylation reagents were removed by centrifugation, flowthrough was discarded. Centrifugal units were washed once with denaturing buffer, flowthrough was discarded. Three more buffer exchanges were made to FASP digestion buffer (50 mM ABC), 200 µL of FASP digestion buffer each time. Proteins were digested overnight in 100 µL FASP digestion buffer with a 1:50 enzyme-to-sample (*w/w*) ratio using Pierce, trypsin protease MS-grade (Thermo Fisher Scientific, Vilnius, Lithuania) or Pierce, chymotrypsin protease (TLCK treated), MS-grade (Thermo Fisher Scientific, Vilnius, Lithuania). Centrifugal units with digestion reaction mixture were incubated in a 37 °C water bath Sub36 (Grant Instruments, Shepreth, UK) for 16 h. After digestion, the flowthrough was collected into new collection tubes, the centrifugal filter units were rinsed twice with 50 µL FASP digestion buffer, the flowthrough was collected. The solvent from the recovered peptide fraction was evaporated using a vacuum dryer Speed Vac SC110 (Thermo Savant, Waltham, MA, USA). Dried peptides were resuspended in 100 µL 50% methanol and vacuum dried two more times to remove volatile salts completely. Dried peptides were redissolved in 0.1% formic acid and Hi3 PhosB standard peptide mixture (Waters Corporation, Milford, MA, USA) was added to yield a final concentration of 0.2 pmol/µL. Prepared samples were analyzed by LC-MS/MS.

### 2.7. Liquid Chromatography and Mass Spectrometry

Liquid chromatography (LC) separation of trypsin cleaved peptides was performed with ACQUITY UPLC I-Class System (Waters Corporation, Milford, MA, USA). Peptides were separated on a reversed-phase analytical column ACQUITY UPLC Peptide BEH C18 Column 300 Å, 1.7 µm, 2.1 mm × 150 mm (Waters Corporation, Milford, MA, USA) at a flow rate of 40 µL/min The isocratic gradient 5% solvent B was set until 2.5 min, then a linear gradient from 5% to 35% solvent B was set until 50 min, followed by a linear gradient from 35 to 85% solvent B until 51 min (solvent A: 0.1% formic acid, solvent B: 100% acetonitrile and 0.1% formic acid). The analytical column temperature was set to 40 °C.

The LC was coupled online through an ESI ionization source with Synapt G2 mass spectrometer (Waters Corporation, Milford, MA, USA). Data was acquired using MassLynx version 4.2 software (Waters Corporation, Milford, MA, USA) in positive ion mode. LC-MS data was collected using data-independent acquisition (DIA) mode MSE. The source/TOF conditions were set as follows: resolution mode, capillary voltage 2.6 kV, sampling cone voltage 40 V, extraction cone voltage 4 V, source temperature 120 °C, desolvation gas flow 800 L/h at 450 °C. During spectral data acquisition, the trap collision energy of the mass spectrometer was ramped from 18 to 35 eV for high-energy scans in MSE mode, for low-energy scans collision energy was disabled. The mass range was set to 50–2000 Da with a scan time set to 0.5 s. A reference compound (2 ng/μL in 50% acetonitrile, 0.1% formic acid) leucine enkephalin (Waters Corporation, Milford, MA, USA) was co-infused continuously at a 7 µL/min flow rate and scanned every 45 s as a reference for accurate mass measurements (reference mass: m/z 556.2771).

### 2.8. Data Processing and Protein Identification

For peptide and protein identification raw data files were processed using ProteinLynx Global SERVER (PLGS) version 3.0.3 (Waters Corporation, Milford, MA, USA). The following parameters were used to generate peak lists: low and elevated energy thresholds, 135 and 20 counts, respectively; reference mass correction window, 0.25 Da at 556.2771 Da/e. Processed data was analyzed using the following parameters: trypsin or chymotrypsin was selected as a primary digest reagent, one missed cleavage was permitted, carbamidomethylation of cysteines was set as a fixed modification, deamidation of asparagine and glutamine, oxidation of methionine were set as variable modifications. Minimal identification criteria included 2 fragment ions per peptide, 5 fragment ions per protein and a minimum of 1 peptide per protein. The false discovery rate (FDR) was set to 2%.

### 2.9. Cloning of GH81 Gene

Gene sequence variants coding PsLam81A (native enzyme sequence with *C*-terminal 6×His tag), PsLam81AΔCBM56 (truncated enzyme sequence without CBM56 domain with *C*-terminal 6×His tag) were cloned from genomic DNA of *Paenibacillus* sp. GKG using Phusion Plus PCR Master Mix (Thermo Fisher Scientific) and designed primers PsLam81A and PsLam81AΔCBM56 (Table S1). The PCR products were stained with ROTI Load DNA stain 1 SYBR Green (Carl-Roth, Karlsruhe, Germany) and ran in a 1% agarose gel electrophoresis. Bands corresponding to target gene sizes were excised from gel and plasmid DNA was extracted from gel slices using GeneJET Gel Extraction Kit (Thermo Fisher Scientific). Purified PCR products were cloned into a pLATE31 cloning vector using aLICator LIC Cloning and Expression Kit 3 (*C*-terminal His-tag) (Thermo Fisher Scientific, Vilnius, Lithuania). *E. coli* strain DH5α (Fermentas, Vilnius, Lithuania) cells were transformed with reaction mixtures and spread on LB agar plates supplemented with 75 µg/mL ampicillin. pDNA was extracted and purified using the ZR Plasmid Miniprep kit (Zymo Research, Irvine, CA, USA). pDNA was sequenced using LIC sequencing primers (Table S1) by the Sanger method (Macrogen Europe, Amsterdam, The Netherlands). Two additional sequencing primers PsLam81A-mid were designed for native enzyme sequence coding gene to obtain whole gene sequence coverage.

### 2.10. Expression and Purification of Recombinant Proteins

The recombinant pLATE31-PsLam81A and pLATE31-PsLam81AΔCBM56 plasmids were transformed into *E. coli* strain BL21(DE3) (Novagen, Darmstadt, Germany) cells. Transformed bacterium were cultured in 20 mL LB medium supplemented with 75 µg/mL ampicillin until it reached OD 0.6–0.8 at 600 nm. Expression of recombinant protein was induced with 0.5 mM IPTG by incubating at 20 °C for 24 h. Bacterial cells were harvested by centrifugation at 7200× *g* for 30 min at 4 °C. Collected cells were resuspended in 5 mL 1 × PBS pH 7.0, sonicated and centrifuged at 30,100× *g* for 30 min at 4 °C. The supernatant was loaded onto a 1 mL HiTrap Chelating HP column (GE Healthcare, Uppsala, Sweden) that had been pre-equilibrated with 10 column volumes of 1 × PBS pH 7.0. Column was washed with 5 column volumes of 28 mM imidazole in 1 × PBS pH 7.0. The recombinant protein was eluted with 400 mM imidazole in 1 × PBS pH 7.0. The collected recombinant protein fraction was desalted on a 5 mL Sephadex G25 column (GE Healthcare, Uppsala, Sweden) using 1 × PBS pH 7.0. The molecular weight of the recombinant protein was evaluated using 12% sodium dodecyl sulfate-polyacrylamide gel electrophoresis (SDS-PAGE) using Prestained Protein Ladder (10–170 kDa) (Thermo Fisher Scientific, Vilnius, Lithuania). Protein bands were visualized using Coomassie Brillant Blue G-250 (Fluka, Buchs, Switzerland) staining. The protein concentration was estimated using Pierce Coomassie (Bradford) Protein Assay Kit (Thermo Fisher, Rockford, IL, USA).

### 2.11. Enzyme Assay

The activity of the recombinant PsLam81A and PsLam81AΔCBM56 enzymes was determined using the DNS method by determining the reduced sugars released during the hydrolysis of polysaccharides as described previously [20], with modifications. DNS reagent contained 10 g/L 3,5-dinitrosalicylic acid, 300 g/L sodium potassium tartrate and 16 g/L NaOH. The hydrolysis reaction was terminated by immediately transferring of tubes to a boiling water bath for 5 min. After the hydrolysis reaction the sample was centrifuged at 16,000× *g* for 2 min, then a sample was mixed with DNS reagent at a sample-to-DNS reagent ratio (1:5, v/v). The mixture was heated for 5 min in a boiling water bath, then cooled down for 5 min in an ice bath, then left for 5 min at 22 °C for the sample temperature to equilibrate. 100 µL of the reaction mixture were transferred to a round bottom microplate and absorbance at 540 nm was measured. The one unit of endo-1,3-β-glucanase activity is determined as the enzyme amount required to produce the amount of reducing sugar equal to 1 µmol of glucose per min of reaction time. Measurements were performed at least in triplicate and expressed as mean ± standard deviation (SD).

### 2.12. Characterization of Biochemical Properties

The enzymatic activity of recombinant PsLam81A was determined using laminarin as a substrate. The optimal pH was determined by measuring the activity at pH ranging between pH 4.0 and 10.0 at 40 °C for 15 min. The reaction mixture (100 µL) contained 10 µg/mL enzyme and 0.9% (*w/v*) laminarin in 50 mM of various buffers: sodium citrate buffer (pH 3.0–6.5), sodium phosphate buffer (pH 6.5–8.0), Tris-HCl buffer (pH 8.0–9.0), glycine-NaOH buffer (pH 9.0–10.0).

The effect of temperature on PsLam81A and PsLam81AΔCBM56 activity was determined in a reaction mixture (100 µL) containing 5 µg/mL enzyme and 0.9% (*w/v*) laminarin in 50 mM sodium phosphate buffer pH 7.0 at different temperatures ranging from 10 °C to 80 °C for 15 min.

The effect of temperature on the stability of PsLam81A was determined by incubating the enzyme in 1 × PBS pH 7.0 at different temperatures ranging from 4 °C to 60 °C for 15 or 60 min. After incubation activity was determined in a reaction mixture (100 µL) containing 10 µg/mL enzyme and 0.9% (*w/v*) laminarin in 50 mM sodium phosphate buffer pH 7.0 incubated at 40 °C for 15 min.

### 2.13. Determination of Substrate Specificity

The mode of action of PsLam81A and PsLam81AΔCBM56 was determined using laminari-oligosaccharides (laminaribiose, laminaritriose, laminaritetraose, laminaripentaose, laminarihexaose (Table 1) as substrates. Reaction mixtures (50 µL) contained 25 µg/mL enzyme and 0.9% (*w/v*) laminari-oligosaccharide substrate in 50 mM sodium phosphate buffer pH 7.0 and were incubated at 40 °C for 2 h, during control reactions the enzymes were absent. The substrate specificity of PsLam81A and PsLam81AΔCBM56 was determined using various polysaccharides: laminarin, yeast β-glucan, curdlan, pachyman, barley β-glucan (Table 1). Reaction mixtures (100 µL) contained 25 µg/mL enzyme and 0.9% (*w/v*) polysaccharide substrate in 50 mM sodium phosphate buffer pH 7.0 and were incubated at 40 °C for 18 h, during control reactions the enzymes were absent. No temperature or chemical pre-treatment was applied to laminari-oligosaccharides or polysaccharides prior to the hydrolysis reaction. The substrates were dissolved or suspended in appropriate buffers by mixing at 1000 rpm at 22 °C for 30 min.

### 2.14. Analysis of Hydrolysis Products

Samples collected at different times of hydrolysis reaction were immediately boiled for 5 min, and then analyzed by thin layer chromatography (TLC). TLC was performed as described [21], with slight modifications. Briefly, samples were spotted on TLC Aluminum Sheets coated with silica gel matrix and developed in butan-1-ol/acetic acid/water (2:1:1, *v/v*) solvent. TLC results were visualized by *p*-anisaldehyde. Developed silica plates were dipped into dye solution (*p*-anisaldehyde/99.8% acetic acid/96% ethanol/96% sulfuric acid 0.54:0.54:50:1.9 *v/v*/*v/v*) for a few seconds and heated above heating plate for a few minutes until spots corresponding to hydrolysis products appeared.

### 2.15. Nucleotide Sequence Accession Number

The *Paenibacillus* sp. GKG family GH81 endo-β-1,3-glucanase gene coding sequence was deposited in GenBank under accession no. OP093985. The *Paenibacillus* sp. GKG 16S rRNA gene sequence was deposited in GenBank under accession no. OP081028.

## 3. Results and Discussion

### 3.1. Identification of PsLam81A Coding Gene

The bacterium (latter named, *Paenibacillus* sp. GKG) was isolated from the water pond sample based on its ability to produce hydrolysis zones on an agar plate containing insoluble yeast cell wall substrate (Figure S1). The isolate was identified using 16S rRNA gene sequence analysis, which showed the highest similarity (99%) with *Paenibacillus* sp. B01 (accession no. CP045802). To identify hydrolytic enzymes, *Paenibacillus* sp. GKG was cultivated in a liquid mineral medium supplemented with yeast cell walls. The supernatant was collected, concentrated, dialyzed and analyzed by LC-MS/MS proteomics in order to identify the extracellular hydrolytic enzymes. The protein sequences (1,671,348 entries) retrieved from UniProt using the *Paenibacillus* genus keyword were used as a reference database. The proteomic analysis revealed that 710 proteins from the database had one to 35 peptides matched to their amino acid sequences. The clustering of these proteins using a 90% identity allowed the identification of 163 proteins in the cell-free culture medium of *Paenibacillus* sp. GKG. The majority of identified proteins were not related to carbohydrate degradation. A manually curated list of 24 proteins selected as CAZymes can be found in Supplementary materials (Table S2). The selection was made based on available assigned EC numbers and protein descriptions. According to the published data, a GH81 family includes a low number of the characterized bacterial enzymes, therefore, a putative glycoside hydrolase belonging to family 81 had 29% sequence coverage and was selected for further analysis to broaden our knowledge about this group of enzymes.

**Figure 1:**
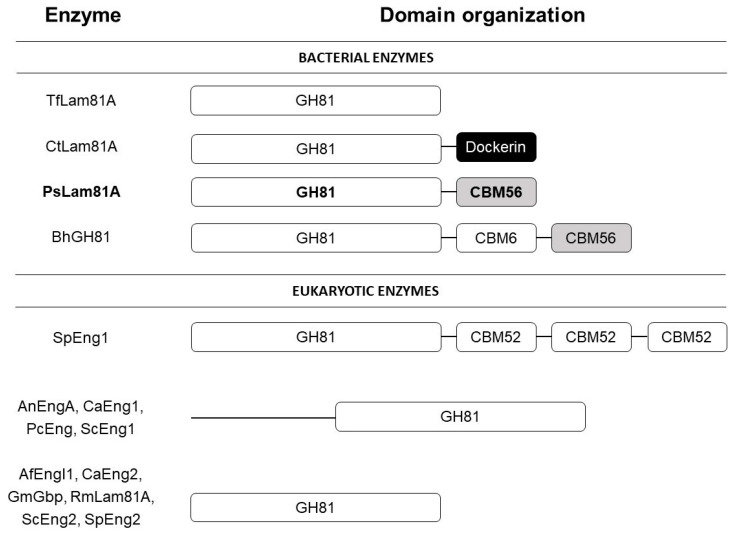
Modular schematics of arrangement of domains in characterized enzymes from GH81 family according to CAZy and InterPro databases. PsLam81A enzyme reported in this study is marked in bold. Domains with a white background are referenced in the CAZy database and are found using a Pfam search. Domains with a grey background are referenced in the CAZy database, but are not found using a Pfam search. Domain with a black background is found using a Pfam search, but is not referenced in the CAZy database. Modular boundaries in the picture are unified for illustration purposes and do not represent the exact sizes of domains.

Up to date, three bacterial β-1,3-glucanases from the GH81 family have been characterized: TfLam81A [4], BH026 [5], and CtLam81A [3]. The two later ones have their crystal structures determined as well. Several bacterial β-1,3-glucanases have different additional domains, but none of them shows the same domain organization as PsLam81A reported in this study (Figure 1). According to the bioinformatic analysis, PsLam81A consisted of two domains: a catalytic GH81 and a CBM56 module, BH026 has a three-domain organization: catalytic GH81, CBM6 and CBM56 modules, CtLam81A has two-domain organization and TfLam81A has only catalytic domain.

There are 11 characterized members from the GH81 family of eukaryotic origin microorganisms, so far. Only one GH from *Schizosaccharomyces pombe* (SpEng1) has three CBM52 domains besides a catalytic domain [22]. AnEngA from *Aspergillus nidulans* [23], CaEng1 from *Candida albicans* [24], PcEng from *Pneumocystis carinii* [25], and ScEng1 from *Saccharomyces cerevisiae* [26] has a catalytic domain and a hypothetical non-cytoplasmic domain. AfEngl1 from *Aspergillus fumigatus* [27], CaEng2 from *Candida albicans* [24], GmGbp from *Glycine max* [28], RmLam81A from *Rhizomucor miehei* [29], ScEng2 from *Saccharomyces cerevisiae* [22], and SpEng2 from *Schizosaccharomyces pombe* [22] have only catalytic domains.

### 3.2. Cloning, Expression and Purification of Recombinant PsLam81A and PsLam81AΔCBM56 Enzymes

**Figure 2:**
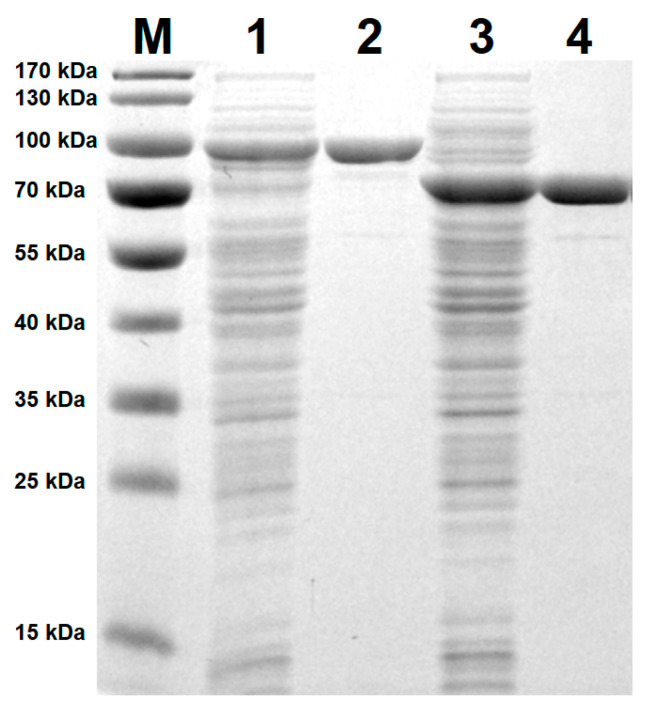
SDS-PAGE analysis of induced and purified recombinant PsLam81A and PsLam81AΔCBM56 proteins. Lane M—Molecular mass, lane 1—soluble protein fraction of induced *E. coli* strain BL21 (DE3) cells carrying pLATE31-PsLam81A plasmid, lane 2—purified PsLam81A protein, lane 3—soluble protein fraction of induced *E. coli* strain BL21 (DE3) cells carrying pLATE31-PsLam81AΔCBM56 plasmid, lane 4—purified PsLam81AΔCBM56 protein. Soluble protein fractions were obtained by centrifugation of cell lysates at 16,000× *g* at 4 °C for 20 min. Proteins were separated by SDS-PAGE (12% separating and 4.0% stacking gels). Gels were developed in Coomassie Brilliant Blue G-250 dye.

The two gene variants of selected glycoside hydrolase from family 81 (coding full PsLam81A and without carbohydrate-binding module PsLam81AΔCBM56 proteins) were cloned into a pLATE31 plasmid, expressed in *E. coli* strain BL21(DE3) cells. Both enzymes were soluble independently of induction at either 20 °C or 30 °C temperatures (Figure S2). Small amounts of target proteins were detected in the induction medium suggesting a low-efficiency secretion. The presence of *N*-terminal signal peptide sequence (1–23 aa) in the PsLam81A protein was predicted by SignalP 6.0 server [30]. Both enzymes with *C*-terminal 6 × His tags were purified successfully using immobilized metal ion affinity chromatography and desalted by gel permeation chromatography. The obtained recombinant enzymes were analyzed by SDS-PAGE (Figure 2). The molecular weights of both enzymes PsLam81A and PsLam81AΔCBM56 were comparable to the ones deduced from theoretical amino acid sequences (see Supplementary Materials). However, a resolution was too low to precisely determine the presence or absence of the signal peptides.

For that reason the purified recombinant PsLam81A enzyme was analyzed by LC-MS/MS. The PsLam81A protein was digested with two proteases separately—trypsin and chymotrypsin. The sequence coverage obtained after tryptic and chymotryptic digestion was 86% and 78%, respectively (Figures S3 and S4). Overlayed sequences of both digestions yielded in 96% coverage of the recombinant protein sequence. The sequence had one uncovered region (1–27 amino acid (aa)), which included a predicted signal peptide sequence (1–23 aa), determined using SignalP 6.0 server [30]. The absence of peptides from this region in both digests indicated that the signal peptide was cleaved off in the *E. coli* cells. Only a small amount of recombinant enzymes was detected in the cell-free culture medium (Figure S2). As the major part of induced proteins remained in the cell and had its signal sequence removed it is probable that proteins remained located in a periplasmic space of *E. coli* cells. Earlier findings support the prediction that the presence of native *Paenibacillus* sp. GKG signal peptide results in periplasmic secretion of PsLam81A and PsLam81AΔCBM56 in *E. coli* BL21(DE3) cells. Hence, the endoglucanase EG5B (a full-length enzyme with a native signal peptide) from *Paenibacillus* sp. IHB B 3084 was transported to the periplasm of *E. coli* BL21(DE3) cells [31]. Moreover, the xylanase XynA from *Paenibacillus* sp. DG-22 heterologously produced in *E. coli* M15 (pREP4) cells was also located in the periplasm when the host cells were carrying the plasmid pXA8 with a gene encoding a full-length protein including a native signal peptide, or in the cytoplasm when host cells were carrying the plasmid pQE60-XynA with a truncated gene (the sequence encoding a native signal peptide was removed) [32].

### 3.3. Effect of pH and Temperature on the Activity of PsLam81A and PsLam81AΔCBM56

**Figure 3:**
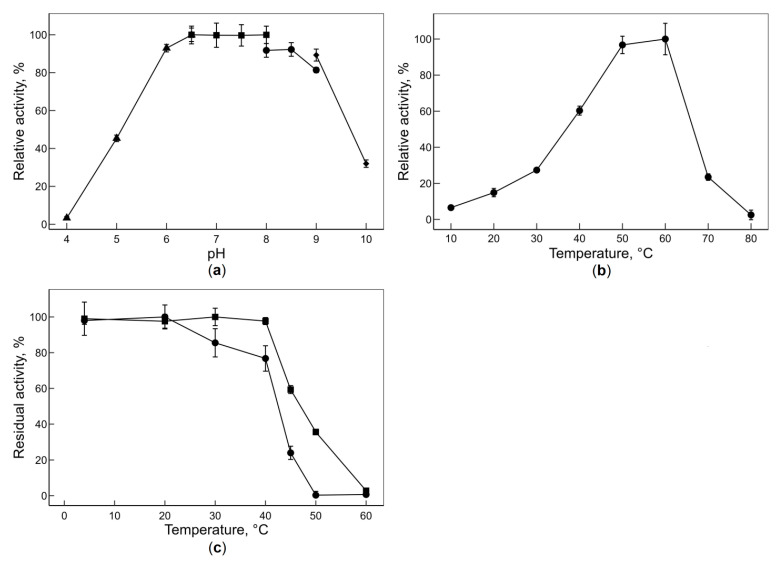
Effect of pH and temperature on the activity of PsLam81A enzyme. (**a**) Effect of pH on enzyme activity in sodium citrate buffer (triangles), sodium phosphate buffer (squares), Tris-HCl buffer (circles), glycine-NaOH buffer (diamonds). (**b**) Effect of temperature on enzyme activity. (**c**) Effect of temperature on enzyme stability after incubation at indicated temperatures for 15 min (squares), 60 min (circles). The bars represent SD.

The purified recombinant protein PsLam81A was active towards yeast cell walls. Therefore, the effect of pH on the activity and the effect of temperature on enzyme activity and stability were tested. The enzyme showed a broad optimum pH range exhibiting 97% of the maximum activity within the pH range of 6.5 and 8.0 and 80% of the maximum activity within the pH range of 6.0 and 9.0 (Figure 3a). Similar optimum pH ranges with 80% of the maximum activity were reported for two previously characterized bacterial GH81 hydrolases—pH range from 5.5 to 8.0 for CtLam81A from *Clostridium thermocellum* (*Acetivibrio thermocellus*) [3] and pH range from 5.5 to 10.0 for TfLam81A from *Thermobifida fusca* [4].

The highest activity of the enzyme (PsLam81A) was recorded at 60 °C (Figure 3b). In comparison, the reported optimal temperature for CtLam81A was 75 °C [3] and 50 °C for TfLam81A [4]. However, the temperature stability of the enzyme (PsLam81A) was low (Figure 3c). After 15 min of incubation at temperatures below 40 °C enzyme retained 97% of maximum activity, whereas at 45 °C only 59% of maximum activity was retained. Moreover, after 60 min incubation the enzyme retained 77% of the maximum activity at 40 °C temperature and showed no activity after incubation at temperatures above 50 °C. Generally, the previously characterized members of the GH81 family have been more thermostable. In comparison, CtLam81A retained 93% of maximum activity after incubation for 60 min at 75 °C [3] and TfLam81A retained approximately 90% of its maximum activity after incubation for 18 h at 50 °C [4].

Literature research revealed that the removal of CBM modules can result in either improved [33,34] or decreased [35,36] thermal stability of an enzyme. Moreover, improved and decreased thermal stability can be observed for different truncation variants for the same enzyme. For example, truncated endo-β-1,4-xylanase rXTMAΔNC with all four (2 × CBM22, 2 × CBM9) CBM modules removed shows lower thermal stability, whereas rXTMAΔC with only two out of four CBM modules (2 × CBM22) removed shows higher thermal stability compared to native enzyme rXTMA where all four CBM modules are present [37]. In this study, we had observed a slight improvement of thermal stability of the PsLam81A enzyme when the CBM56 module was removed (the enzyme PsLam81AΔCBM56). After 60 min of incubation at 45 °C, PsLam81A retained 24.0 ± 2.7% of maximum activity, PsLam81AΔCBM56 retained 35.6 ± 1.0% of maximum activity and after 60 min incubation at 50 °C PsLam81A retained no detectable activity, when PsLam81AΔCBM56 showed a trace activity. The fusion of the homologous CBM56 module with chitosanases improved the thermal stability of fusion enzymes compared to native ones [38]. However, the thermal stability enhancing effect was different for the two chitosanases, hence, CBM56-GsCsn46A had improved thermal stability only at 35 °C temperature, while CBM56-BaCsn46A was more stable in the 40–55 °C temperature range. These observations on the CBM56 module effect on temperature stability supports the pattern that every enzyme and CBM combination is case-dependent and there is no general rule to conclude whether CBM module presence will improve or decrease the thermal stability of a truncated or fused enzyme.

We have selected the temperature of 40 °C for the substrate specificity reactions (Section 3.4). This is a compromise between an increasing reaction rate (Figure 3b) and the decreasing thermal stability of the enzyme (Figure 3c) due to increasing reaction temperature.

### 3.4. Substate Specificity of PsLam81A and PsLam81AΔCBM56

Various polysaccharides (Table 1) were assayed for hydrolysis by recombinant PsLam81A and PsLam81AΔCBM56. The specific activity of each enzyme for the polysaccharide substrates was calculated by quantifying the amount of reducing sugars released during the hydrolysis reaction. The polysaccharides that were hydrolyzed by both enzyme variants were laminarin, yeast β-glucan, curdlan and pachyman (Table 2).

The full-length enzyme variant (PsLam81A) showed the highest activity towards laminarin, followed by yeast β-glucan and curdlan with very similar specific activities and was the least active on pachyman. This data is in agreement with previously published data on the specificity of the GH81 hydrolases [3,4,5,22,26,27,39]. PsLam81A exhibited lower specific activity towards pachyman compared to laminarin (∼4.1-fold lower activity) and curdlan (∼1.8-fold lower activity). These ratios are different to previously reported data. A ∼8.5-fold lower specific activity of TfLam81A towards pachyman compared to laminarin [4], and a > 15-fold lower activity of BH026 on pachyman relative to laminarin as well as a > 10-fold lower activity on pachyman versus curdlan [40] have been reported previously.

The truncated variant of an enzyme (PsLam81AΔCBM56) hydrolyzed the same substrates as a full-length enzyme. The specific activity of PsLam81AΔCBM56 towards the β-1,6 branches containing polysaccharides such as laminarin and yeast β-glucan was similar or higher in comparison to a full-length counterpart. However, PsLam81AΔCBM56 had lower specific activity in the presence of the β-1,3-linear β-glucans (curdlan and pachyman). The importance of CBM modules on curdlan hydrolysis by the GH81 family enzyme was also demonstrated in a previous study [40]. Moreover, the removal of a CBM1 module from the GH45 hydrolase affected an activity towards polysaccharides [35]. The current understanding of such activity enhancement profile by CBM on insoluble substrates, but not on soluble substrates is observed because of the increased local enzyme concentration on the surface of the polysaccharide particles via CBM binding [40]. However, there is evidence that CBM removal can decrease the enzymatic activity simultaneously on both soluble and insoluble substrates. The activity of endo-β-1,3-glucanase (TnLam16A) containing two CBM4 modules decreased by approximately 70% on laminarin and pachyman, and 60% on curdlan after removal of both CBM modules. Moreover, the enzyme with removed the *N*-terminal CBM4 module only showed a higher activity loss towards laminarin than curdlan and pachyman [41].

The deletion of the *C*-terminal module CBM56 from the BH026 enzyme resulted in ∼3-fold lower activity, and the subsequent removal of the second module CBM6 resulted in ∼6-fold lower activity on curdlan relative to a native BH026 enzyme [40]. In this study, the deletion of the CBM56 module from PsLam81A resulted in a ∼2.4-fold lower activity towards curdlan. These results show that the CBM56 module is functional in the full-length enzyme variant PsLam81A and has an effect on a specific activity of the PsLam81A enzyme when insoluble polysaccharides are used as a substrate.

The polysaccharides that were not hydrolyzed by any of the tested enzyme variants were barley β-glucan, sugar beet β-glucan, pustulan, carboxymethyl cellulose, microcrystalline cellulose (Table 2). The majority of these substrates were reported to be non-hydrolyzable by GH81 enzymes, and only a minor hydrolytic activity towards barley β-glucan [4,27], pustulan [4,26] and carboxymethyl cellulose [3,4,26,27] was detected.

**Figure 4:**
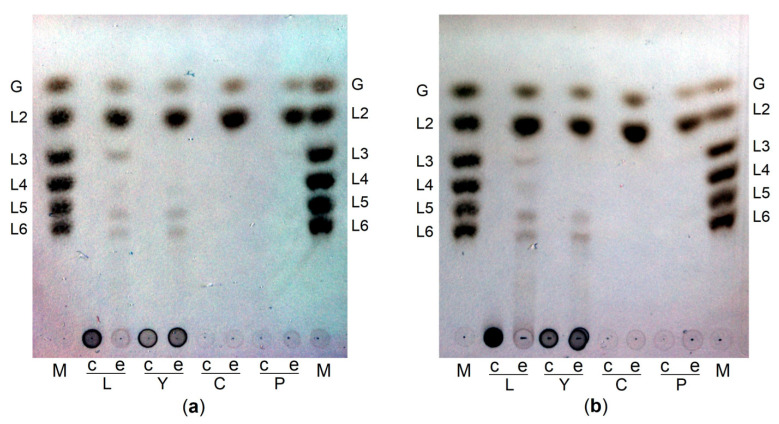
TLC analysis of hydrolysis products. Hydrolysis of laminarin (L), yeast β-glucan (Y), curdlan (C), and pachyman (P) by PsLam81A (**a**) and PsLam81AΔCBM56 (**b**). Carbohydrate standards (M): glucose (G), laminaribiose (L2), laminaritriose (L3), laminaritetraose (L4), laminaripentaose (L5) and laminarihexaose (L6). Reactions were carried out in the presence (e) and absence (c) of the enzyme.

Analysis of hydrolysis products of laminarin, yeast β-glucan, curdlan and pachyman by both PsLam81A and PsLam81AΔCBM56 showed similar profiles (Figure 4a,b) and laminaribiose was the main product. In the presence of linear β-glucans (curdlan, pachyman) the enzymes yielded laminaribiose as the main product and glucose as the second one. The same pattern was observed in the case of β-1,6-branches containing polysaccharides (laminarin and yeast β-glucan). However, some other hydrolysis products corresponding to compounds with a higher degree of polymerization (DP) were detected. Two spots near L6 standard were detected for both branched polysaccharides. Similar differences between laminarin and curdlan hydrolysis products after prolonged hydrolysis reaction were reported earlier [5]. Most likely, these additional spots are linear β-1,3 glucan oligosaccharides with β-1,6 branches appearing due to incomplete hydrolysis limited by β-1,6 bonds. The additional difference (hydrolysis products corresponding to L3 and L4 present in laminarin, but not present in yeast β-glucan) between these two polysaccharides containing β-1,6 branches is possibly visible due to different terminal monosaccharides composition. Yeast β-glucan contains glucose in the β-1,3 glucan backbone, while laminarin can be terminated with mannitol or glucose [8]. Therefore, laminarin may contain longer terminal oligosaccharides that are non-hydrolyzable due to the presence of mannitol in comparison to oligosaccharides with the same DP containing only glucose.

**Figure 5:**
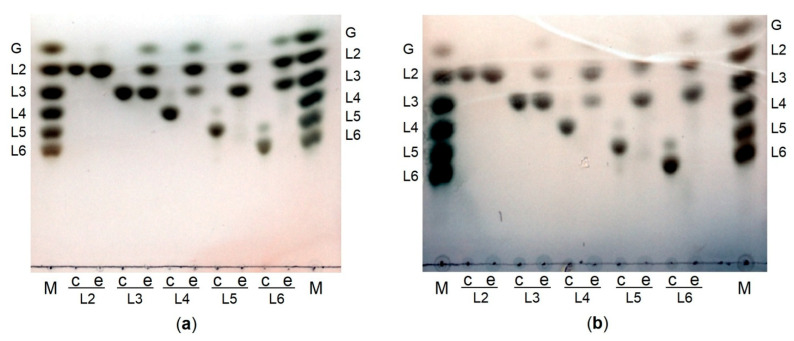
TLC analysis of hydrolysis products. Hydrolysis of laminari-oligosaccharides (laminaribiose (L2), laminaritriose (L3), laminaritetraose (L4), laminaripentaose (L5), laminarihexaose (L6) by PSLam81A (**a**) and PsLam81AΔCBM56 (**b**). Carbohydrate standards (M): glucose (G), laminaribiose (L2), laminaritriose (L3), laminaritetraose (L4), laminaripentaose (L5) laminarihexaose (L6). Reactions were carried out in the presence (e) and absence (c) of the enzyme.

The analysis of hydrolysis products of laminari-oligosaccharides with PsLam81A and PsLam81AΔCBM56 showed that both enzymes were able to degrade laminarihexaose (L6), laminaripentaose (L5), laminaritetraose (L4), laminaritriose (L3) down to laminaribiose (L2) and glucose (Figure 5a,b).

Laminaritetraose, laminaripentaose and laminarihexaose were completely hydrolyzed after 2 h incubation while laminaritriose was not degraded completely. However, the 18 h prolonged incubation of PsLam81A and PsLam81AΔCBM56 with curdlan and pachyman resulted in glucose and laminaribiose without the accumulation of laminaritriose (Figure 4a,b). These observations indicate that tested enzymes have lower activity towards shorter oligosaccharides compared to longer ones. This is in agreement with earlier reported data on the endo-hydrolytic activity of the GH81 hydrolases when a decrease in activity correlated with the decrease of the length of oligosaccharide [22,27,28,39]. Laminaribiose was not degraded by PsLam81A and PsLam81AΔCBM56, indicating that laminaritriose was a minimal substrate for both enzymes. There is one other endo-glucanase belonging to the GH81 family that is reported to use laminaritriose as a minimal substrate [5]. Moreover, the disappearance of laminaritriose during laminarihexaose hydrolysis indicates that laminaritriose can be used as a substrate by β-1,3-glucanase Lam81A from *Thermobifida fusca* [4]. Also, the decreasing proportion of laminaritriose with increasing curdlan hydrolysis time indicates that laminaritriose is a substrate of the enzyme from *Clostridium thermocellum* [3]. However, some of the reported data is not straightforward, because a low activity on laminaritriose is not always considered to be sufficient to conclude that laminaritriose is a substrate for the GH81 family glucanases [22,28].

All this data showed that PsLam81A as several other hydrolases from the GH81 family can act on laminaritriose as a minimal substrate. In addition, the identical results for both enzymes PsLam81A and PsLam81AΔCBM56 indicated that the CBM56 module does not have an effect on the substrate specificity for oligosaccharides.

## 4. Conclusions

The endo-β-1,3-glucanase PsLam81A from *Paenibacillus* sp. GKG was identified, expressed heterologously and characterized as a representative of the GH81 family. PsLam81A hydrolyzed β-1,3 glycosidic bonds containing polysaccharides such as laminarin, yeast β-glucan, curdlan, and pachyman down to laminaribiose as the main hydrolysis product. The minimal laminari-oligosaccharide substrate hydrolyzed by PsLam81A was laminaritriose. PsLam81A characterized in this study has a unique domain organization compared to other characterized GHs of the family 81. PsLam81A contains a single CBM56 domain that ensures more efficient hydrolysis of water-insoluble substrates. Collected data encourage further studies focused on the application of PsLam81A for hydrolysis of the yeast wall under industrial conditions.

## Figures and Tables

**Table 1:** Substrates used in this study.

Substrate	Glycosidic Bond	Reference Number	Source
Laminaribiose	β-1,3	O-LAM2	Megazyme
Laminaritriose	β-1,3	O-LAM3	Megazyme
Laminaritetraose	β-1,3	O-LAM4	Megazyme
Laminaripentaose	β-1,3	O-LAM5	Megazyme
Laminarihexaose	β-1,3	O-LAM6	Megazyme
Laminarin	β-1,3; β-1,6	L9634	Sigma-Aldrich
Carboxymethyl cellulose	β-1,4	419281	Sigma-Aldrich
Microcrystalline cellulose	β-1,4	435236	Sigma-Aldrich
Curdlan	β-1,3	P-CURDL	Megazyme
Pachyman	β-1,3	P-PACHY	Megazyme
Pustulan	β-1,6	YP15423	Carbosynth
Barley β-glucan	β-1,3; β-1,4	P-BGBL	Megazyme
Yeast β-glucan	β-1,3; β-1,6	P-BGYST	Megazyme
Sugar beet β-glucan	β-1,2	P-BGLU12	Megazyme

**Table 2:** Substrate specificity of PsLam81A and PsLam81AΔCBM56.

Substrate	U/mg		U × 10^9^/mol	
	PsLam81A	PsLam81AΔCBM56	PsLam81A	PsLam81AΔCBM56
Laminarin	108.8 ± 1.7	125.0 ± 2.8	10.1 ± 0.2	10.1 ± 0.2
Yeast β-glucan	47.7 ± 1.6	64.1 ± 2.4	4.4 ± 0.1	5.1 ± 0.2
Curdlan	48.9 ± 4.4	23.5 ± 1.2	4.5 ± 0.4	1.9 ± 0.1
Pachyman	26.6 ± 2.0	8.7 ± 0.7	2.5 ± 0.2	0.7 ± 0.1
Other ^1^	ND	ND	ND	ND

^1^ barley β-glucan, sugar beet β-glucan, pustulan, carboxymethyl cellulose, microcrystalline cellulose. ND—not detected.

## Data Availability

The mass spectrometry proteomics data have been deposited to the ProteomeXchange Consortium via the PRIDE [42] partner repository with the dataset identifier PXD036565.

## Supplementary Materials

The following supporting information can be downloaded at: https://www.mdpi.com/article/10.3390/microorganisms10101930/s1, Figure S1: Extracellular hydrolases producing bacteria growing on solid mineral medium containing water-insoluble yeast cell wall substrate as a carbon source; Figure S2: SDS-PAGE analysis of recombinant PsLam81A and PsLam81AΔCBM56 proteins induced at different temperatures; Figure S3: The graphical representation of PsLam81A sequence coverage (86%) obtained after tryptic digestion; Figure S4: The graphical representation of PsLam81A sequence coverage (78%) obtained after chymotryptic digestion; Table S1: Primers used in this study; Table S2: List of CAZymes manually selected from *Paenibacillus* sp. GKG proteins identified by analyzing secretome by LC-MS/MS proteomics.

## References

[1] Caseiro C., Dias J.N.R., de Andrade Fontes C.M.G., Bule P. (2022). From Cancer Therapy to Winemaking: The Molecular Structure and Applications of β-Glucans and β-1, 3-Glucanases. Int. J. Mol. Sci..

[2] Henrissat B. (1991). A Classification of Glycosyl Hydrolases Based on Amino Acid Sequence Similarities. Biochem. J..

[3] Kumar K., Correia M.A.S., Pires V.M.R., Dhillon A., Sharma K., Rajulapati V., Fontes C.M.G.A., Carvalho A.L., Goyal A. (2018). Novel Insights into the Degradation of β-1,3-Glucans by the Cellulosome of Clostridium Thermocellum Revealed by Structure and Function Studies of a Family 81 Glycoside Hydrolase. Int. J. Biol. Macromol..

[4] McGrath C.E., Wilson D.B. (2006). Characterization of a *Thermobifida fusca* β-1,3-Glucanase (Lam81A) with a Potential Role in Plant Biomass Degradation. Biochemistry.

[5] Pluvinage B., Fillo A., Massel P., Boraston A.B. (2017). Structural Analysis of a Family 81 Glycoside Hydrolase Implicates Its Recognition of β-1,3-Glucan Quaternary Structure. Structure.

[6] Boraston A.B., Bolam D.N., Gilbert H.J., Davies G.J. (2004). Carbohydrate-Binding Modules: Fine-Tuning Polysaccharide Recognition. Biochem. J..

[7] Qin Z., Yang D., You X., Liu Y., Hu S., Yan Q., Yang S., Jiang Z. (2017). The Recognition Mechanism of Triple-Helical β-1,3-Glucan by a β-1,3-Glucanase. Chem. Commun..

[8] Read S.M., Currie G., Bacic A. (1996). Analysis of the Structural Heterogeneity of Laminarin by Electrospray-Ionisation-Mass Spectrometry. Carbohydr. Res..

[9] Legentil L., Paris F., Ballet C., Trouvelot S., Daire X., Vetvicka V., Ferrières V. (2015). Molecular Interactions of β-(1→3)-Glucans with Their Receptors. Molecules.

[10] Lipke P.N., Ovalle R. (1998). Cell Wall Architecture in Yeast: New Structure and New Challenges. J. Bacteriol..

[11] Klis F.M., Mol P., Hellingwerf K., Brul S. (2002). Dynamics of Cell Wall Structure in *Saccharomyces cerevisiae*. FEMS Microbiol. Rev..

[12] Orlean P. (2012). Architecture and Biosynthesis of the *Saccharomyces Cerevisiae* Cell Wall. Genetics.

[13] Ma L., Lu Y., Yan H., Wang X., Yi Y., Shan Y., Liu B., Zhou Y., Lü X. (2020). Screening of Cellulolytic Bacteria from Rotten Wood of Qinling (China) for Biomass Degradation and Cloning of Cellulases from Bacillus Methylotrophicus. BMC Biotechnol..

[14] Chen F., Ye J., Sista Kameshwar A.K., Wu X., Ren J., Qin W., Li D.-W. (2020). A Novel Cold-Adaptive Endo-1,4-β-Glucanase From Burkholderia Pyrrocinia JK-SH007: Gene Expression and Characterization of the Enzyme and Mode of Action. Front. Microbiol..

[15] Yan Q., Yang H., Jiang Z., Liu E., Yang S. (2018). A Novel Thermostable β-1,3-1,4-Glucanase from Thermoascus Aurantiacus and Its Application in Oligosaccharide Production from Oat Bran. Carbohydr. Res..

[16] Borchani C., Fonteyn F., Jamin G., Paquot M., Blecker C., Thonart P. (2014). Enzymatic Process for the Fractionation of Baker’s Yeast Cell Wall (*Saccharomyces cerevisiae*). Food Chem..

[17] Woo T.H., Cheng A.F., Ling J.M. (1992). An Application of a Simple Method for the Preparation of Bacterial DNA. BioTechniques.

[18] Weisburg W.G., Barns S.M., Pelletier D.A., Lane D.J. (1991). 16S Ribosomal DNA Amplification for Phylogenetic Study. J. Bacteriol..

[19] Erde J., Loo R.R.O., Loo J.A. (2014). Enhanced FASP (EFASP) to Increase Proteome Coverage and Sample Recovery for Quantitative Proteomic Experiments. J. Proteome Res..

[20] Wood I.P., Elliston A., Ryden P., Bancroft I., Roberts I.N., Waldron K.W. (2012). Rapid Quantification of Reducing Sugars in Biomass Hydrolysates: Improving the Speed and Precision of the Dinitrosalicylic Acid Assay. Biomass Bioenergy.

[21] Qin Z., Yan Q., Yang S., Jiang Z. (2016). Modulating the Function of a β-1,3-Glucanosyltransferase to That of an Endo-β-1,3-Glucanase by Structure-Based Protein Engineering. Appl. Microbiol. Biotechnol..

[22] Martín-Cuadrado A.-B., Fontaine T., Esteban P.-F., del Dedo J.E., de Medina-Redondo M., del Rey F., Latgé J.P., de Aldana C.R.V. (2008). Characterization of the Endo-β-1,3-Glucanase Activity of S. cerevisiae Eng2 and Other Members of the GH81 Family. Fungal Genet. Biol..

[23] Szilágyi M., Kwon N.-J., Dorogi C., Pócsi I., Yu J.-H., Emri T. (2010). The Extracellular β-1,3-Endoglucanase EngA Is Involved in Autolysis of Aspergillus Nidulans: Aspergillus Nidulansβ-1,3-Endoglucanase EngA. J. Appl. Microbiol..

[24] Esteban P.F., Ríos I., García R., Dueñas E., Plá J., Sánchez M., de Aldana C.R.V., del Rey F. (2005). Characterization of the CaENG1 Gene Encoding an Endo-1,3-β-Glucanase Involved in Cell Separation in Candida Albicans. Curr. Microbiol..

[25] Kutty G., Davis A.S., Ma L., Taubenberger J.K., Kovacs J.A. (2015). Pneumocystis Encodes a Functional Endo-β-1,3-Glucanase That Is Expressed Exclusively in Cysts. J. Infect. Dis..

[26] Baladrón V., Ufano S., Dueñas E., Martín-Cuadrado A.B., del Rey F., Vázquez de Aldana C.R. (2002). Eng1p, an Endo-1,3-β-Glucanase Localized at the Daughter Side of the Septum, Is Involved in Cell Separation in *Saccharomyces cerevisiae*. Eukaryot. Cell.

[27] Fontaine T., Hartland R.P., Beauvais A., Diaquin M., Latge J.-P. (1997). Purification and Characterization of an Endo-1,3-Beta-Glucanase from Aspergillus Fumigatus. Eur. J. Biochem..

[28] Fliegmann J., Montel E., Djulić A., Cottaz S., Driguez H., Ebel J. (2005). Catalytic Properties of the Bifunctional Soybean β-Glucan-Binding Protein, a Member of Family 81 Glycoside Hydrolases. FEBS Lett..

[29] Zhou P., Chen Z., Yan Q., Yang S., Hilgenfeld R., Jiang Z. (2013). The Structure of a Glycoside Hydrolase Family 81 Endo-β-1,3-Glucanase. Acta Crystallogr. D Biol. Crystallogr..

[30] Teufel F., Almagro Armenteros J.J., Johansen A.R., Gíslason M.H., Pihl S.I., Tsirigos K.D., Winther O., Brunak S., von Heijne G., Nielsen H. (2022). SignalP 6.0 Predicts All Five Types of Signal Peptides Using Protein Language Models. Nat. Biotechnol..

[31] Dhar H., Kasana R.C., Gulati A. (2015). Heterologous Expression and Characterization of Detergent Stable Endoglucanase EG5B from Paenibacillus Sp. IHB B 3084. J. Mol. Catal. B Enzym..

[32] Lee T.H., Lim P.O., Lee Y.-E. (2007). Cloning, Characterization, and Expression of Xylanase A Gene from Paenibacillus Sp. DG-22 in Escherichia Coli. J. Microbiol. Biotechnol..

[33] Cheng R., Cheng L., Wang L., Fu R., Sun X., Li J., Wang S., Zhang J. (2019). Characterization of an Alkali-Stable Xyloglucanase/Mixed-Linkage β-Glucanase Pgl5A from Paenibacillus Sp. S09. Int. J. Biol. Macromol..

[34] Wang Y., Yuan H., Wang J., Yu Z. (2009). Truncation of the Cellulose Binding Domain Improved Thermal Stability of Endo-β-1,4-Glucanase from Bacillus Subtilis JA18. Bioresour. Technol..

[35] Couturier M., Feliu J., Haon M., Navarro D., Lesage-Meessen L., Coutinho P.M., Berrin J.-G. (2011). A Thermostable GH45 Endoglucanase from Yeast: Impact of Its Atypical Multimodularity on Activity. Microb. Cell Factories.

[36] Winterhalter C., Heinrich P., Candussio A., Wich G., Liebl W. (1995). Identification of a Novel Cellulose-Binding Domain the Multidomain 120 KDa Xylanase XynA of the Hyperthermophilic Bacterium Thermotoga Maritima. Mol. Microbiol..

[37] Verjans P., Dornez E., Segers M., Van Campenhout S., Bernaerts K., Beliën T., Delcour J.A., Courtin C.M. (2010). Truncated Derivatives of a Multidomain Thermophilic Glycosyl Hydrolase Family 10 Xylanase from Thermotoga Maritima Reveal Structure Related Activity Profiles and Substrate Hydrolysis Patterns. J. Biotechnol..

[38] Lin S., Qin Z., Chen Q., Fan L., Zhou J., Zhao L. (2019). Efficient Immobilization of Bacterial GH Family 46 Chitosanase by Carbohydrate-Binding Module Fusion for the Controllable Preparation of Chitooligosaccharides. J. Agric. Food Chem..

[39] Ma J., Qin Z., Zhou P., Wang R., Yan Q., Jiang Z., Yang S. (2022). Structural Insights into the Substrate Recognition and Catalytic Mechanism of a Fungal Glycoside Hydrolase Family 81 β-1,3-Glucanase. Enzyme Microb. Technol..

[40] Hettle A., Fillo A., Abe K., Massel P., Pluvinage B., Langelaan D.N., Smith S.P., Boraston A.B. (2017). Properties of a Family 56 Carbohydrate-Binding Module and Its Role in the Recognition and Hydrolysis of β-1,3-Glucan. J. Biol. Chem..

[41] Zverlov V.V., Volkov I.Y., Velikodvorskaya G.A., Schwarz W.H. (2001). The Binding Pattern of Two Carbohydrate-Binding Modules of Laminarinase Lam16A from Thermotoga Neapolitana: Differences in β-Glucan Binding within Family CBM4. Microbiology.

[42] Perez-Riverol Y., Bai J., Bandla C., García-Seisdedos D., Hewapathirana S., Kamatchinathan S., Kundu D.J., Prakash A., Frericks-Zipper A., Eisenacher M. (2022). The PRIDE Database Resources in 2022: A Hub for Mass Spectrometry-Based Proteomics Evidences. Nucleic Acids Res..

