# Supplementary material for: Characterization of Paenibacillus sp. GKG Endo-β-1, 3-Glucanase, a Member of Family 81 Glycoside Hydrolases
Source: Microorganisms. 2022 Sep 28;10(10):1930. doi: 10.3390/microorganisms10101930 (PMC9609564; doi:10.3390/microorganisms10101930)
Supplement: Supplementary file 1 [file microorganisms-10-01930-s001.zip › microorganisms-1935338-supplementary.pdf]

Supplementary material

Characterization of *Paenibacillus* sp. GKG Endo-β-1,3-Glucanase, a Member of Family 81 Glycoside Hydrolases

Gediminas Plakys[1,2,*], Renata Gasparavičiūtė[1], Justas Vaitekūnas[1], Rasa Rutkienė[1] and Rolandas Meškys[1,*]

[1] Department of Molecular Microbiology and Biotechnology, Institute of Biochemistry, Life Sciences Center, Vilnius University, Sauletekio 7, Vilnius, LT-10257, Lithuania

[2] R&D Department, Roquette Amilina, AB, J. Janonio 12, Panevezys, LT-35101, Lithuania

* Correspondence: gediminas.plakys@bchi.stud.vu.lt (G.P.); rolandas.meskys@bchi.vu.lt (R.M.)

(**a**)

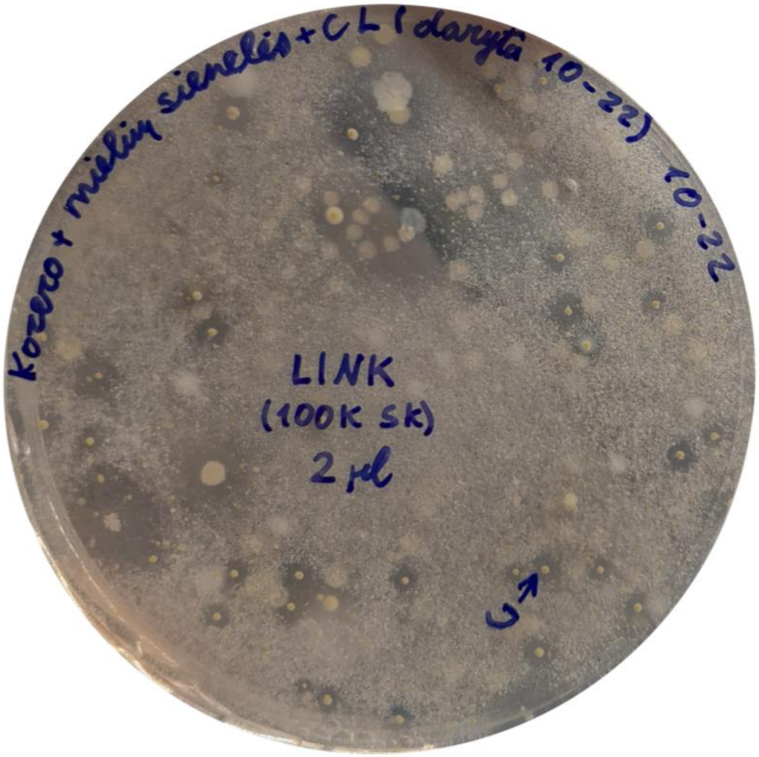

(**b**)

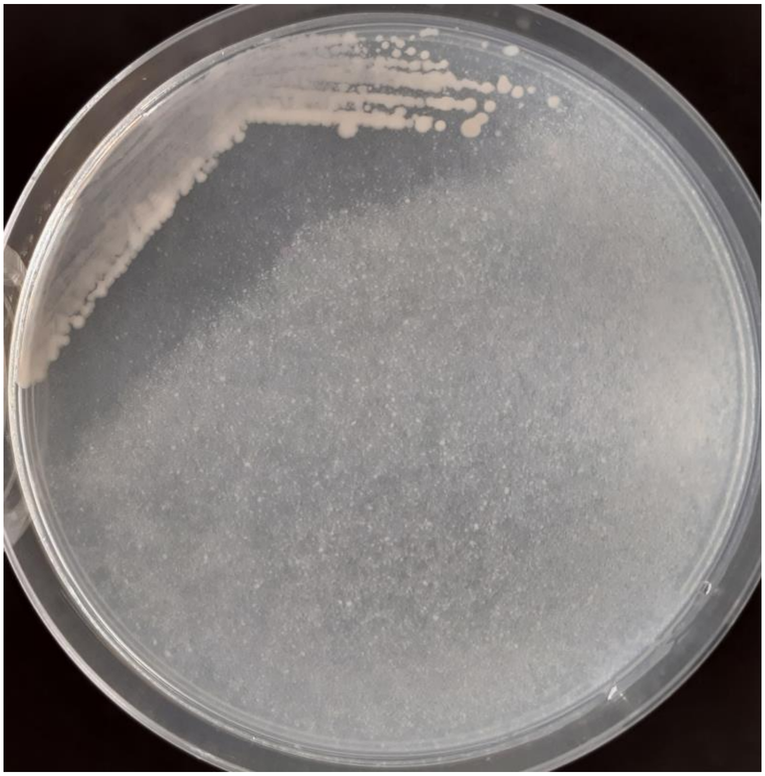

Supplementary Figure S1. Extracellular hydrolases producing bacteria growing on solid mineral medium containing water-insoluble yeast cell wall substrate as a carbon source. (**a**) A typical screening plate acquired after plating of yeast cell wall enriched water pond sample, (**b**) Isolated *Paenibacillus* sp. GKG bacterium. Clear hydrolysis zone around colonies shows the presence of extracellular hydrolases.

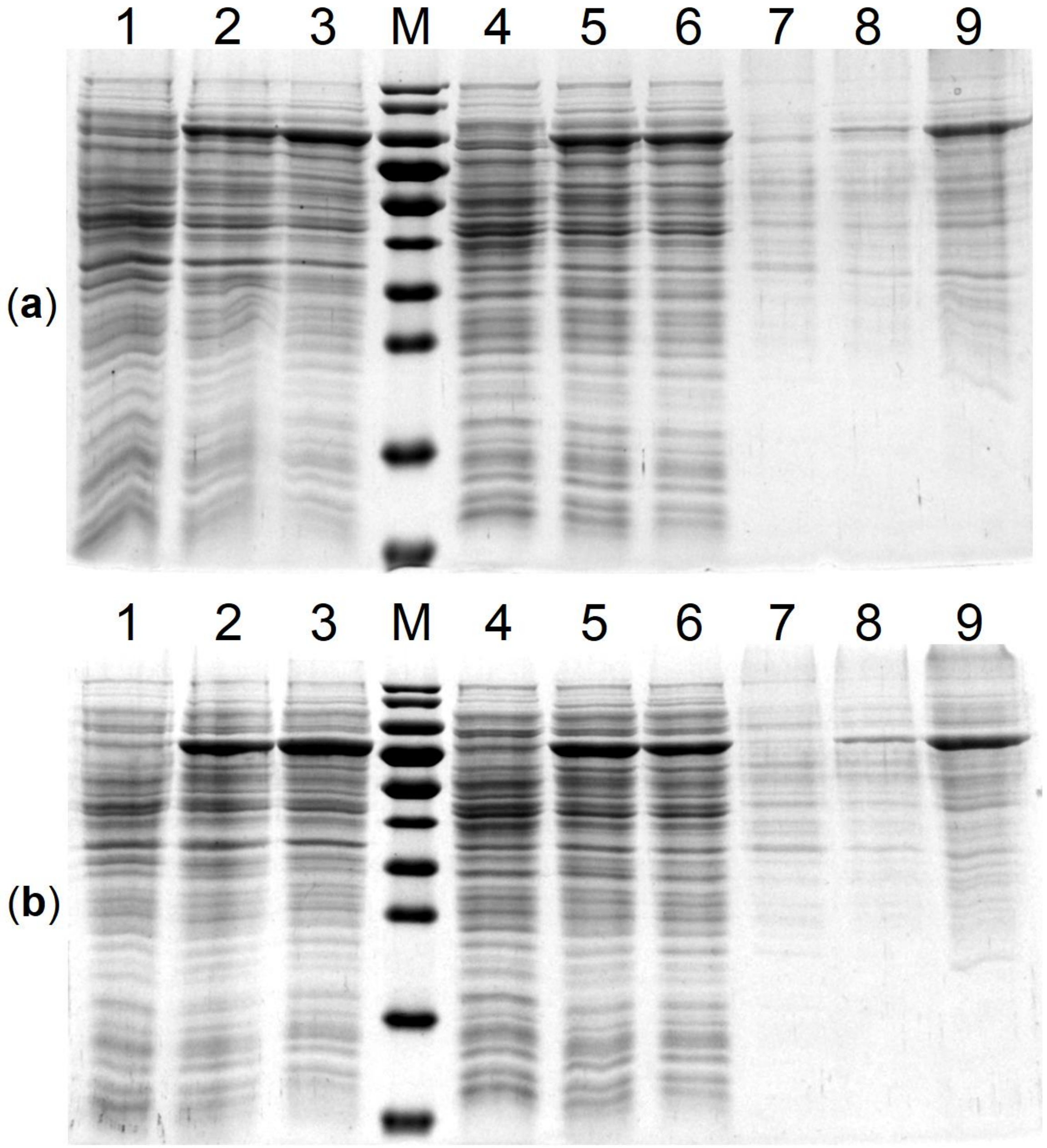

Supplementary Figure S2. SDS-PAGE analysis of recombinant PsLam81A and PsLam81AΔCBM56 proteins induced at different temperatures. *E. coli* strain BL21(DE3) cells expressing (**a**) PsLam81A and (**b**) PsLam81AΔCBM56 were induced at 20 °C and 30 °C for 24 hours. Lane M – Molecular mass marker (10–170 kDa, Thermo Fisher Scientific). Lane 1 – cell lysate induced at 30 °C (without target gene plasmid (wp)), lane 2 – cell lysate induced at 20 °C (plasmid with target gene present (p)), 3 – lysate of cells induced at 30 °C (p), 4 – soluble protein fraction* of cell lysate induced at 30 °C (wp), 5 – soluble protein fraction of cell lysate induced at 20 °C (p), 6 – soluble protein fraction of cell lysate induced at 30 °C (p), 7 – 50× concentrated culture medium proteins** separated from cells induced at 30 °C (wp), 8 – 50× concentrated culture medium proteins separated from cells induced at 20 °C (p), 9 – 50× concentrated culture medium proteins separated from cells induced at 30 °C (p).

*soluble protein fraction was obtained by centrifugation of cell lysate at 16,000× *g* at 4 °C for 20 minutes.

**proteins were concentrated by incubating culture medium for 10 minutes in a boiling water bath and collecting precipitated proteins by centrifugation at 16,000× *g* for 20 minutes.

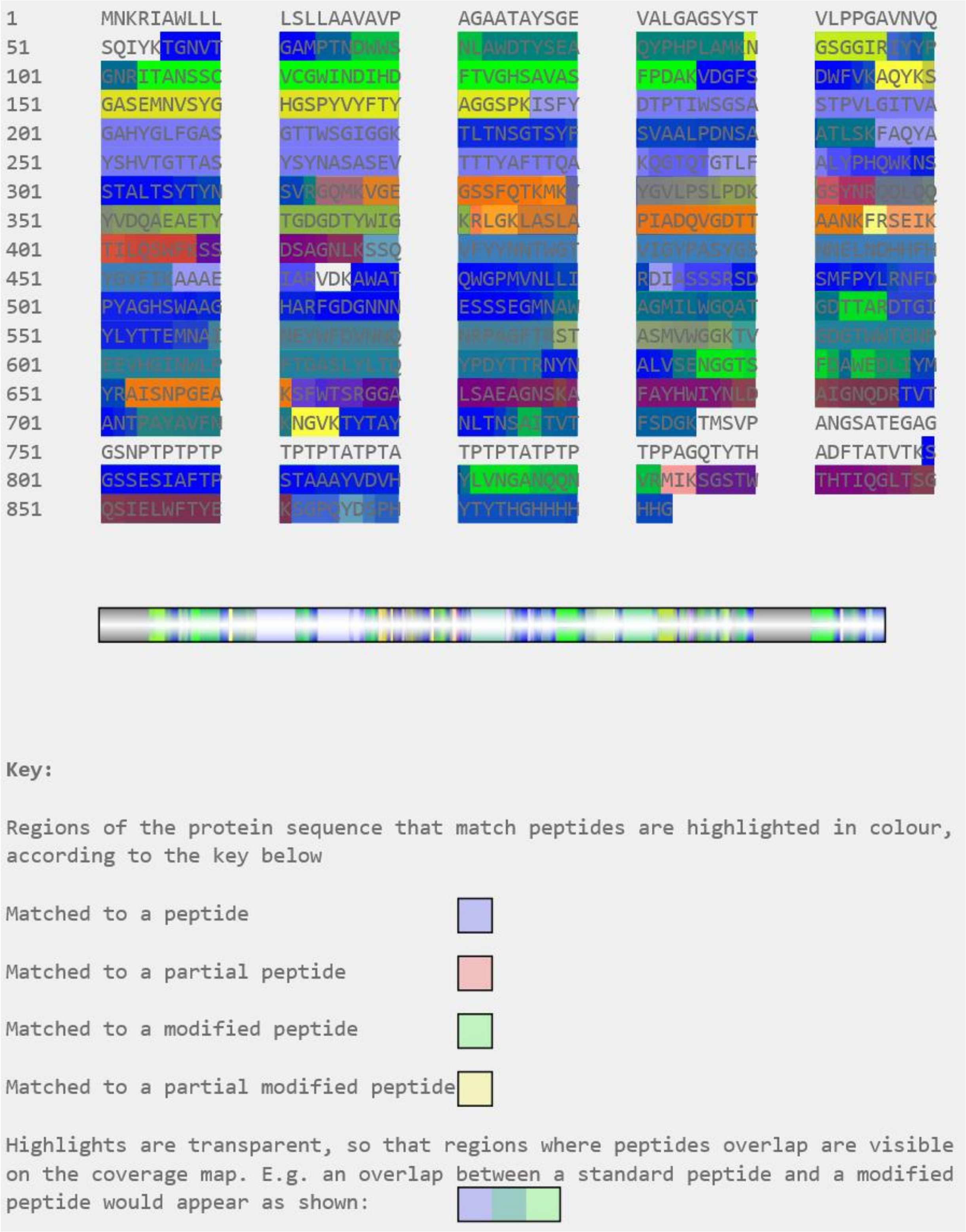

Supplementary Figure S3. The graphical representation of PsLam81A sequence coverage (86%) obtained after tryptic digestion.

1	MNKRIAWLLL	LSLLAAVAVP	AGAATAYSGE	VALGAGSYST	VLPPGAVNVQ
51	SQIYKTGNVT	GAMPTNDWWS	NLAWDTYSEA	QYPHPLAMKN	GSGGIRIYYP
101	GNRITANSSC	VCGWINDIHD	FTVGHSAVAS	FPDAKVDGFS	DWFVKAQYKS
151	GASEMNVSYG	HGSPYVYFTY	AGGSPKISFY	DTPTIWSGSA	STPVLGITVA
201	GAHYGLFGAS	GTTWSGIGGK	TLTNSGTSYF	SVAALPDNSA	ATLSKFAQYA
251	YSHVTGTTAS	YSYNASASEY	TTTYAFTTQA	KQGTQTGTLF	ALYPHQWKNS
301	STALTSYTYN	SVRGQMKVGE	GSSFQTKMKY	YGVLPSLPDK	GSYNRQQLQQ
351	YVDQAEAETY	TGDGDTYWIG	KRLGKLASLA	PIADQVGDTT	AANKFRSEIK
401	TILQSWFKSS	DSAGNLKSSQ	VFYYNNTWGT	VIGYPASYGS	NNELNDHHFH
451	YGYFIKAAAE	IARVDKAWAT	QWGPMVNLLI	RDIASSSRSD	SMFPYLRNFD
501	PYAGHSWAAG	HARFGDGNNN	ESSSEGMNAW	AGMILWGQAT	GDTTARDTGI
551	YLYTTEMNAI	NEYWFDVNNQ	NRPAGFTRST	ASMVWGGKTV	GDGTW[illegible]TGNP
601	EEVHGINWLP	FTGASLYLTQ	YPDYTTRNYN	ALVSENGGTS	FDAWEDLIYM
651	YRAISNPGEA	KSFWTSRGGA	LSAEAGNSKA	FAYHWIYNLD	AIGNQDRTVT
701	ANTPAYAVFN	KNGVKTYTAY	NLTNSAITVT	FSDGKTMSVP	ANGSATEGAG
751	GSNPTPTPTP	TPTPTATPTA	TPTPTATPTP	TPPAGQTYTH	ADFTATVTKS
801	GSSESIAFTP	STAAAYVDVH	YLVNGANQQN	VRMIKSGSTW	THTIQGLTSG
851	QSIELWFTYE	KSGPQYDSPH	YTYTHGHHHH	HHG	

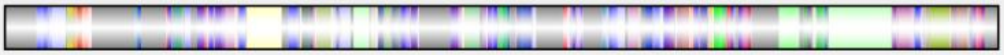

Key:

Regions of the protein sequence that match peptides are highlighted in colour, according to the key below

Matched to a peptide

Matched to a partial peptide

Matched to a modified peptide

Matched to a partial modified peptide

Highlights are transparent, so that regions where peptides overlap are visible on the coverage map. E.g. an overlap between a standard peptide and a modified peptide would appear as shown:

Supplementary Figure S4. The graphical representation of PsLam81A sequence coverage (78%) obtained after chymotryptic digestion.

Supplementary Table S1. Primers used in this study.

Primer	Primer sequence 5′-3′	Source
Fw W001	AGAGTTTGATCMTGGCTC	[18]
Rv W002	GNTACCTTGTTACGACTT	[18]
Fw pJET1.2	CGACTCACTATAGGGAGAGCGGC	Thermo Fisher Scientific
Rv pJET1.2	AAGAACATCGATTTTCCATGGCAG	Thermo Fisher Scientific
Fw PsLam81A	AGAAGGAGATATAACTATGAACAAACGAATCGCCTG	This study
Fw PsLam81AΔCBM56	AGAAGGAGATATAACTATGAACAAACGAATCGCCTG	This study
Fw PsLam81A-mid	GTACGCCTACTCGCATGTGACG	This study
Rv PsLam81A-mid	GTACTGCGTCAGGTAGAGC	This study
Rv PsLam81A	GTGGTGGTGATGGTGATGGCCATGCGTATACGTATAATGC	This study
Rv PsLam81AΔCBM56	GTGGTGGTGATGGTGATGGCCATTGCTGCCGCCCGC	This study

Supplementary Table S2. List of CAZymes manually selected* from *Paenibacillus* sp. GKG proteins identified by analysing secretome by LC-MS/MS proteomics.

Accession	Description	Score	Sequence coverage (%)
A0A5Q2NY17	Family 16 glycosylhydrolase OS=Paenibacillus sp. B01	777	18
A0A2N5N8G8	Endo-1_4-beta-xylanase A OS=Paenibacillus pasadenensis	777	17
A0A218NL06	Chitin-binding protein OS=Paenibacillus pasadenensis	536	28
A0A5Q2NWL3	Chitin-binding protein OS=Paenibacillus sp. B01	536	28
A0A2N5ND26	Chitin binding protein OS=Paenibacillus pasadenensis	536	28
A0A343F0L1	Chitinase OS=Paenibacillus pasadenensis	451	19
A0A5Q2NX09	Chitinase OS=Paenibacillus sp. B01	451	20
A0A2N5N0S9	Chitinase OS=Paenibacillus pasadenensis	451	19
A0A6H2GTK2	Chitinase OS=Paenibacillus sp. UniB2	421	17
A0A6H2H2T3	Chitin-binding protein OS=Paenibacillus sp. UniB2	343	9
A0A5Q2NJK9	Glycoside hydrolase family 81 OS=Paenibacillus sp. B01	255	29
A0A2N5N1W0	Chitin binding protein OS=Paenibacillus pasadenensis	210	21
A0A5Q2NS91	Glucan endo-1_6-beta-glucosidase OS=Paenibacillus sp. B01	182	23
A0A2N5N256	Glycosyl hydrolase_ family 30 OS=Paenibacillus pasadenensis	182	23
A0A6H2H3I3	Glycoside hydrolase family 81 OS=Paenibacillus sp. UniB2	142	9
A0A2N5N8U3	Endo-1_4-beta-xylanase A OS=Paenibacillus pasadenensis	162	25
A0A5Q2NJF9	Family 16 glycosylhydrolase OS=Paenibacillus sp. B01	132	16
A0A2N5N0U5	Beta-glucanase OS=Paenibacillus pasadenensis	132	16
H6CEV7	Beta-1_6-glucanase OS=Paenibacillus sp. Aloe-11	128	16
A0A2N9YZ34	Endoglucanase OS=Paenibacillus polymyxa	122	14
A0A222WSD2	Beta-1_6-glucanase OS=Paenibacillus kribbensis	120	14
A0A5Q2NPP4	Chitinase OS=Paenibacillus sp. B01	95	13
A0A343F0L2	Chitinase OS=Paenibacillus pasadenensis	95	13
A0A2N5NAZ7	Chitinase OS=Paenibacillus pasadenensis	95	13

*Selection was made using result file Paenibacillus_sp_GKG_secreted_proteins_1_result.zip deposited at ProteomeXchange with the dataset identifier PXD036565

>PsLam81A
MNKRIAWLLLLSLLAAVAVPAGAATAYSGEVALGAGSYSTVLPPGAVNVQSQIYKTGNVTGAMPTND
WWSNLAWDTYSEAQYPHPLAMKNGSGGIRIYYPGNRITANSSCVCGWINDIHDFTVGHSAVASFPDA
KVDGFSDWFVKAQYKSGASEMNVSYGHGSPYVYFTYAGGSPKISFYDTPTIWSGSASTPVLGITVAGAH
YGLFGASGTTWSGIGGKTLTNSGTSYFSVAALPDNSAATLSKFAQYAYSHVTGTTASYSYNASASEVTTT
YAFTTQAKQGTQTGTLFALYPHQWKNSSTALTSYTYNSVRGQMKVGEGSSFQTKMKYYGVLPSLPDK
GSYNRQQLQQYVDQAEAETYTGDGDTYWIGKRLGKLASLAPIADQVGDTTAANKFRSEIKTILQSWFK
SSDSAGNLKSSQVFYYNNTWGTVIGYPASYGSNNELNDHHFHYGYFIKAAAEIARVDKAWATQWGP
MVNLLIRDIASSSRSDSMFPYLRNFDPYAGHSWAAGHARFGDGNNNESSSEGMNAWAGMILWGQAT
GDTTARDTGIYLYTTEMNAINEYWFDVNNQNRPAGFTRSTASMVWGGKTVGDGTWWTGNPEEVHGI
NWLPFTGASLYLTQYPDYTTRNYNALVSENGGTSFDAWEDLIYMYRAISNPGEAKSFWTSRGGALSAE
AGNSKAFAYHWIYNLDAIGNQDRTVTANTPAYAVFNKNGVKTYTAYNLTNSAITVTFSDGKTMSVPA
NGSATEGAGGSNPTPTPTPTPTPTATPTATPTPTATPTPTPPAGQTYTHADFTATVTKSGSSESIAFTPST
AAAYVDVHYLVNGANQQNVRMIKSGSTWTHTIQGLTSGQSIELWFTYEKSGPQYDSPHYTYTHGHHH
HHHG

>PsLam81AΔCBM56
MNKRIAWLLLLSLLAAVAVPAGAATAYSGEVALGAGSYSTVLPPGAVNVQSQIYKTGNVTGAMPTND
WWSNLAWDTYSEAQYPHPLAMKNGSGGIRIYYPGNRITANSSCVCGWINDIHDFTVGHSAVASFPDA
KVDGFSDWFVKAQYKSGASEMNVSYGHGSPYVYFTYAGGSPKISFYDTPTIWSGSASTPVLGITVAGAH
YGLFGASGTTWSGIGGKTLTNSGTSYFSVAALPDNSAATLSKFAQYAYSHVTGTTASYSYNASASEVTTT
YAFTTQAKQGTQTGTLFALYPHQWKNSSTALTSYTYNSVRGQMKVGEGSSFQTKMKYYGVLPSLPDK
GSYNRQQLQQYVDQAEAETYTGDGDTYWIGKRLGKLASLAPIADQVGDTTAANKFRSEIKTILQSWFK
SSDSAGNLKSSQVFYYNNTWGTVIGYPASYGSNNELNDHHFHYGYFIKAAAEIARVDKAWATQWGP
MVNLLIRDIASSSRSDSMFPYLRNFDPYAGHSWAAGHARFGDGNNNESSSEGMNAWAGMILWGQAT
GDTTARDTGIYLYTTEMNAINEYWFDVNNQNRPAGFTRSTASMVWGGKTVGDGTWWTGNPEEVHGI
NWLPFTGASLYLTQYPDYTTRNYNALVSENGGTSFDAWEDLIYMYRAISNPGEAKSFWTSRGGALSAE
AGNSKAFAYHWIYNLDAIGNQDRTVTANTPAYAVFNKNGVKTYTAYNLTNSAITVTFSDGKTMSVPA
NGSATEGAGGSNGHHHHHHG
